# A mechanistic physiologically based model to assess the effect of study design and modified physiology on formulation safe space for virtual bioequivalence of dermatological drug products

**DOI:** 10.3389/fphar.2022.1007496

**Published:** 2022-12-01

**Authors:** J. F. Clarke, K. Thakur, S. Polak

**Affiliations:** ^1^ Simcyp Division, Certara UK, Sheffield, United Kingdom; ^2^ Faculty of Pharmacy, Jagiellonian University Medical College, Krakow, Poland

**Keywords:** PBPK absorption model, dermal absorption, bioequivalence, virtual bioequivalence (VBE), safe space, topical formulations, complex drug products, generic drugs

## Abstract

Physiologically based pharmacokinetic (PBPK) models are widely accepted tools utilised to describe and predict drug pharmacokinetics (PK). This includes the use of dermal PBPK models at the regulatory level including virtual bioequivalence (VBE) studies. The current work considers the Topicort^®^ Spray formulation, which contains 0.25% desoximetasone (DSM), as an example formulation. Quantitative formulation composition and *in vitro* permeation testing (IVPT) data were obtained from the public literature to develop a mechanistic model using the multi-phase, multi-layer (MPML) MechDermA IVPT module in the Simcyp Simulator. *In vitro*–*in vivo* extrapolation functionality was used to simulate *in vivo* PK for various scenarios and predict a ‘safe space’ for formulation bioequivalence using the VBE module. The potential effect of vasoconstriction, impaired barrier function, and various dosing scenarios on the formulation safe space was also assessed. The model predicted ‘safe space’ for formulation solubility suggesting that a 50% change in solubility may cause bio-in-equivalence, whereas viscosity could deviate by orders of magnitude and the formulation may still remain bioequivalent. Evaporation rate and fraction of volatile components showed some sensitivity, suggesting that large changes in the volume or composition of the volatile fraction could cause bio-in-equivalence. The tested dosing scenarios showed decreased sensitivity for all formulation parameters with a decreased dose. The relative formulation bioequivalence was insensitive to vasoconstriction, but the safe space became wider with decreased barrier function for all parameters, except viscosity that was unaffected.

## Introduction

Physiologically based pharmacokinetic (PBPK) models are widely accepted tools used to describe and predict drug pharmacokinetics (PK). They combine and integrate information on the biological system of interest (i.e., morpho-physiological parameters and their distribution in various populations) and drug properties (i.e., physicochemical properties, binding, clearance, and formulation characteristics) within a user-defined trial design (i.e., dose amount, type and duration of application, and number of subjects). Such an approach allows for not only simulation of a base scenario but also extrapolation to alternative scenarios and simulation of alternative hypotheses to predict the interplay between the previously listed factors.

During the last few decades, modelling and simulation in general and PBPK modelling specifically have moved from being an academic area of interest to reaching acceptance at the regulatory level (Anand et al., 2022). The increased use of PBPK models throughout the drug development process is reflected in new drug application submissions to regulatory bodies (Zhang et al., 2020; Wu et al., 2021). This includes the use of dermal PBPK models at the regulatory level for various reasons such as supporting alternative bioequivalence (BE) approaches, for example, virtual BE studies (Tsakalozou et al., 2021), defining a safe space for critical quality attributes (CQAs), and extrapolating bioavailability predictions and BE assessments from healthy to diseased populations (Alam, 2021).

The ability to estimate local skin and systemic exposure to xenobiotics after dermal application is essential in developing new dermatological drugs or assessing the potential toxicological liability of chemicals. Historically, animal models may have been used to evaluate dermal drug absorption prior to clinical testing. However, both differences in human and animal physiology as well as ethical concerns over animal testing have spurred the development of *in vitro* and *in silico* methods to help assess dermal drug absorption. Recent advances in mechanistic model development, their acceptance, and potential role have resulted in advanced models available in the literature (Somayaji et al., 2021; Patel et al., 2022). To utilise these models for assessing the bioavailability of dermatological drug products, the development of mechanistic formulation models that can integrate data from *in vitro* characterisation studies is essential. The quantitative composition of a formulation (Q2) and CQAs such as rheology, solubility, and particle and droplet size (Q3) can be assessed to parameterise the model.

The formulation whilst inside its container is called the “primary formulation”. When assessing the properties of a topical formulation, *in vitro* studies are usually performed on the formulation immediately after being dispensed from the container, the so-called “secondary formulation”. For many topical drug products, however, the composition and character of the formulation change rapidly once exposed to the environment due to evaporation and/or absorption of vehicle components. Once this metamorphosis has been completed (or slowed significantly), what remains can be referred to as the “tertiary formulation”, which in many cases will be the formulation type present for the major portion of the absorption window and therefore its properties, as opposed to those of the primary or secondary formulation, may be most important in determining the rate and extent of absorption (Surber and Knie, 2018). Depending on the duration of the metamorphosis phase, it may also be important to simulate this dynamically.

It is usually possible to predict or assume the composition of the tertiary formulation based on the Q2 composition of the primary or secondary formulation by identifying volatile components. However, it is more challenging to do the same for Q3 properties. The use of mechanistic formulation models allows the sensitivity of these Q3 parameters to be estimated.

Comparing the bioavailability of two dermatological drug products can be challenging due to the complexity of their formulations and the potential interplay between skin physiology and the drug/formulation. Also, many drug products are designed to act locally and often do not reach the systemic circulation to a quantifiable extent. Therefore, it can be challenging and inaccurate to assess the bioavailability using plasma as a PK endpoint; however, quantification of drug amounts at the local site is experimentally more difficult. Some methods commonly used for this purpose are tape stripping (Pershing et al., 2003; Cordery et al., 2017), imaging techniques (Caspers et al., 2019; Handler et al., 2021), microdialysis-based approaches (Garcia Ortiz et al., 2011; Incecayir et al., 2011; Bodenlenz et al., 2017), and biopsy (Marks and Dykes, 1994; Undre et al., 2009).

Although each of these methods has its own limitations, data from all the abovementioned techniques are extremely valuable when assessing the PK of dermatological products. Mechanistic PBPK models can have a synergistic relationship with such data by helping to inform experimental design and providing a framework in which to integrate the results from one or more studies; in return, the data are used to inform and verify the model, which can then be used to extrapolate and answer more complex questions, thereby increasing the potential impact of the data.

Many topically applied drug products indicated for skin diseases are intended to act locally. If PK-based approaches such as those described earlier are used to demonstrate BE, usually the pivotal comparison study will be conducted using healthy human skin. However, it is unclear whether changes in physiology in the intended patient population may affect the relative bioavailability of test and reference formulations.

The current work uses the 0.25% Topicort^®^ Spray formulation of desoximetasone (DSM) as an example formulation. It should be noted that the standard method for showing bioequivalence of a DSM formulation, being a corticosteroid, would be a vasoconstrictor assay (FDA, 2015; Oussedik et al., 2017). This work is not intended to represent a realistic regulatory path for this formulation. Topicort^®^ Spray formulation of DSM was used as a case study due to rare data availability on its Q1 and Q2 (qualitative and quantitative composition of inactive ingredients, respectively) and available IVPT data from a patent (Kisak, 2019). The same patent detailed composition data for 62 alternate formulations, presenting IVPT data for 12 of these. However, it was not possible to directly simulate the alternate formulations for which IVPT data were provided due to the lack of information on DSM solubility in the solvents used, but the data can serve as a reference for formulation sensitivity.

The current work aims to investigate sensitivity to changes in formulation, using Topicort^®^ Spray as a reference, and the impact of bioequivalence study trial design such as various dose amounts and modifications of selected physiology parameters on outcomes for various test formulations.

## Methods

Desoximetasone (DSM) is a corticosteroid applied topically to treat various types of rashes and skin diseases such as psoriasis. It is available as a spray, cream, ointment, or gel. The physicochemical properties of DSM were collected from the literature to develop a PBPK model for this compound, as presented in Table 1.

**TABLE 1:** Physicochemical properties of DSM with reference to relevant sources.

Parameter	Value	Reference
Molecular weight (Da)	376.46	ChemSpider (https://www.chemspider.com/Chemical-Structure.4470604.html)
Log *P*	2.35	Hansch et al. (1995)
Hydrogen bond acceptor	4	ACD/ChemSpider (https://www.chemspider.com/Chemical-Structure.4470604.html)
Compound type	Neutral	Anissimov and Roberts (2011)
Water solubility (mg/ml)	0.0421	Anissimov and Roberts (2011)
Blood/plasma ratio	0.76	Anissimov and Roberts (2011)
fu plasma	0.145	Anissimov and Roberts (2011)
fu dermis	0.054	Anissimov and Roberts (2011)
Kdermis/water	0.17	Anissimov and Roberts (2011)
Clearance_iv_ (L/h)	16.95	Pires et al. (2015)

### Topicort^®^ Spray 0.25%

Topicort^®^ Spray is indicated for plaque psoriasis. The quantitative composition of Topicort^®^ Spray was assumed based on the information provided in a patent (Kisak, 2019).

The Q2 (% w/w) for the major constituents of the formulation is 44% mineral oil, 31.4% isopropyl myristate (IPM), and 23.4% isopropyl alcohol (IPA) as presented in Table 2. The spray was assumed to be a solution formulation type, with DSM fully dissolved in both the primary and tertiary formulations.

**TABLE 2:** Quantitative composition (Q2) of Topicort^®^ Spray.

Component	Primary/secondary (% w/w)	Tertiary (% w/w)	Density (g/ml)	Primary/secondary (% v/v)	Tertiary (% v/v)
Desoximetasone	0.25	0.33	1.3	0.16	0.21
Glyceryl oleate	0.9	1.17	1	0.75	1.00
Isopropyl alcohol	23.4	0.00	0.774	25.17	0.00
Isopropyl myristate	31.38	40.96	0.85	30.74	41.08
L-Menthol	0.05	0.07	0.89	0.05	0.06
Mineral oil	44.03	57.47	0.85	43.13	57.64

Utilising data from Table 2 (volume weighting), the density of the primary formulation can be estimated as 0.83 g/ml and that of the tertiary formulation as 0.85 g/ml. When applied in such a thin layer (10 μL/cm^2^), the IPA evaporates within 2–3 min, leaving the tertiary formulation for the remainder of the study. Therefore, if the tertiary formulation composition were to be used from the start of the simulation, no significant difference is seen in the absorption profile (data not shown).

In the simulations, the continuous phase was assumed to consist of only two major tertiary formulation components; therefore, the normalised volume fractions of mineral oil and IPM were 58.39% and 41.61% v/v, respectively. The molar volume of this mixture was calculated to be 415.8 within the Simcyp Simulator formulation toolbox. There was no information publicly available regarding the Q3 characteristics of the formulation; therefore, the viscosity was assumed to be 100 cP, and a sensitivity analysis of this parameter is provided below.

## IVPT simulations

**FIGURE 1:**
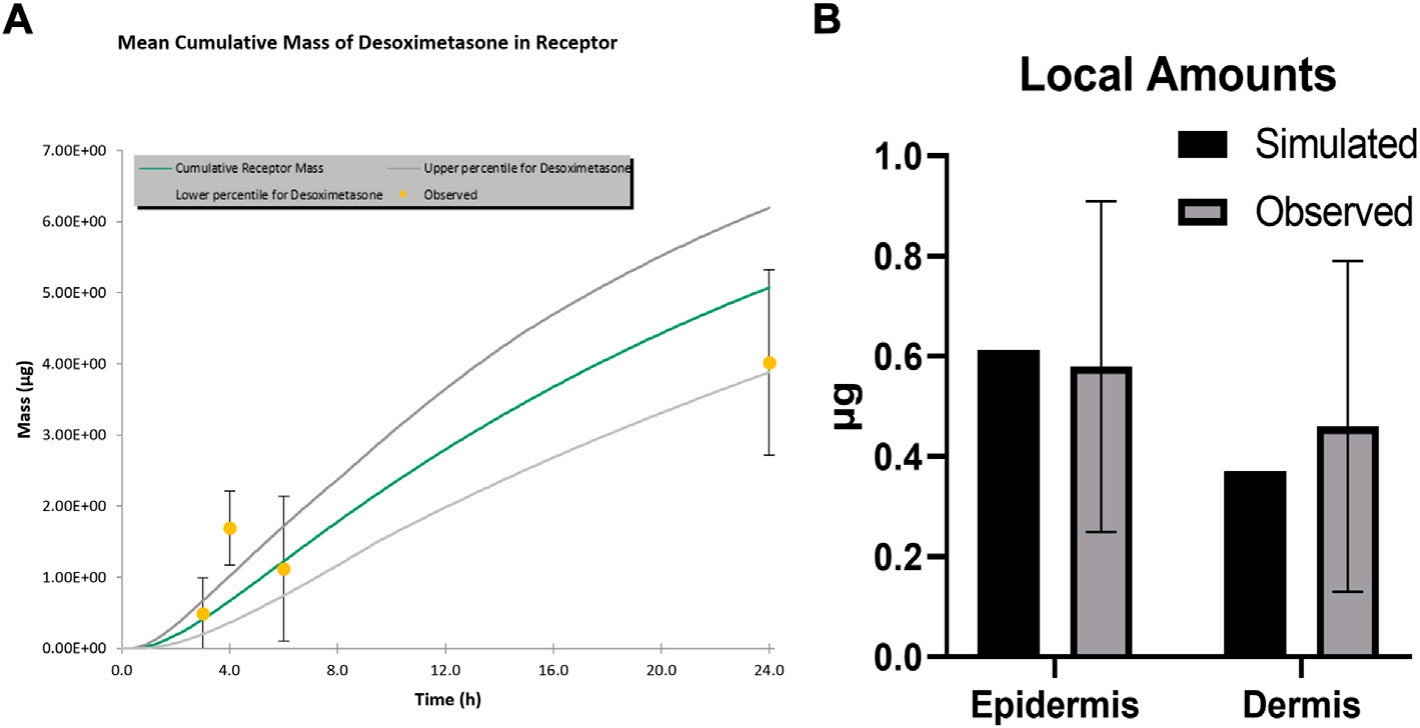
Simulated and observed IVPT results for Topicort^Ⓡ^ Spray 0.25% **(A)** Receptor Profile **(B)** Local amounts.

**FIGURE 2:**
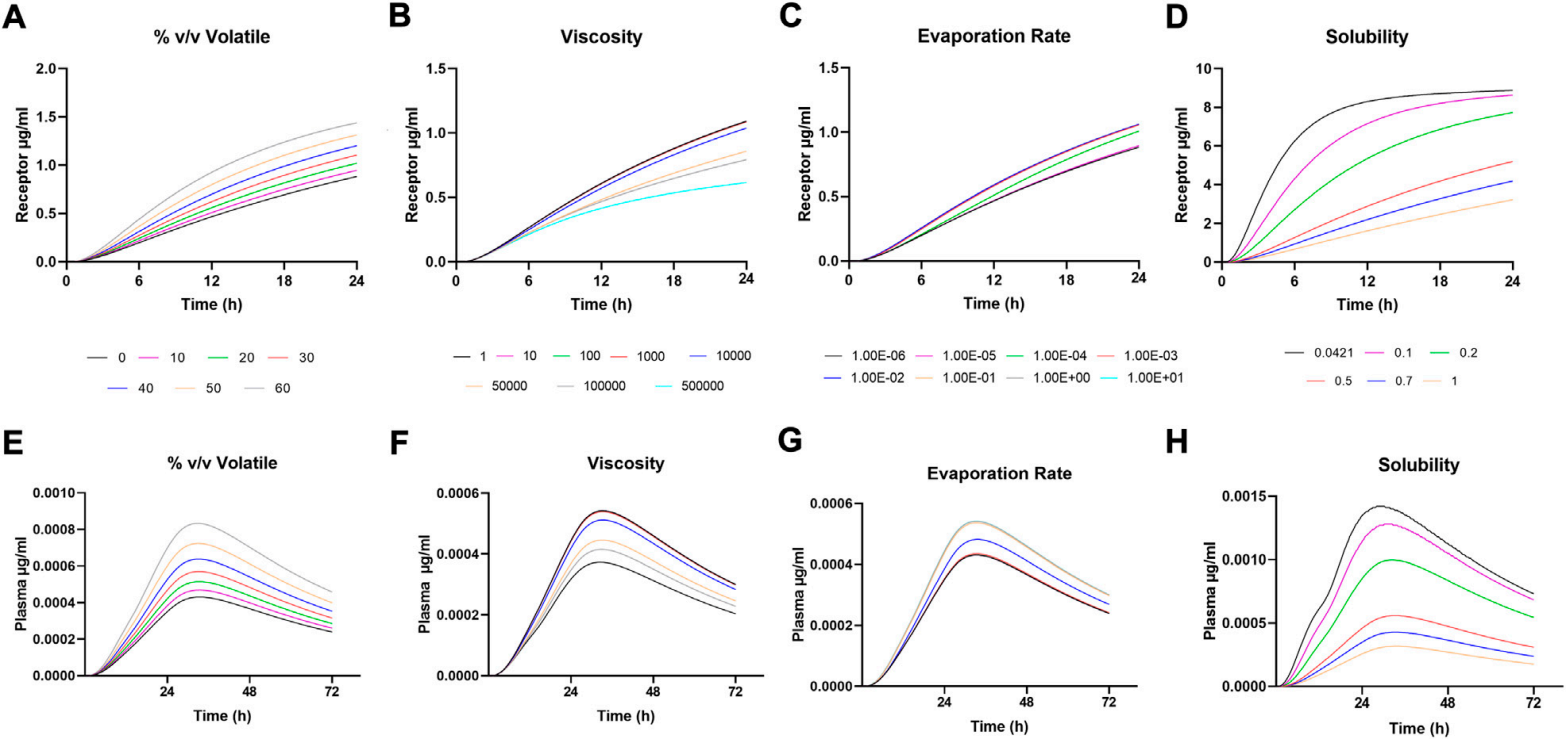
Sensitivity Analysis for **(A-D)** Receptor amounts following IVPT simulation and **(E-H)** Plasma amounts following *in vivo* simulation.

The MPML MechDermA *in vitro* permeation testing (IVPT) module in the Simcyp Simulator version 21 was used to simulate an IVPT study for Topicort^®^ Spray 0.25% (Kisak, 2019). In this study, 5 µL of spray was applied to 0.5 cm^2^ of cadaver skin in a Franz diffusion cell (Moore, 2022). The body site of the skin was unknown; therefore, the abdomen was assumed as the site of application for the simulation. The value of the stratum corneum (SC) lipid to vehicle partition coefficient (*K*_sclip:vehicle_) was manually optimised to match the receptor profile. The parameter value of corneocyte permeability (P_cell_) was manually optimised to match local concentration data. The IVPT methodology stated that three tape strips were taken and discarded; therefore, the first three layers of the stratum corneum were removed in the simulation, and the remaining SC layers were added to the amount in viable epidermis to calculate the amount in the epidermis for comparison to observed data. Observed data were obtained by extracting ‘control’ data from Figures 1, 2, 4, 5 in the patent (Kisak, 2019). The data from Figure 3 in Kisak et al. were excluded as they showed significant disagreement with the other four results.

**FIGURE 3:**
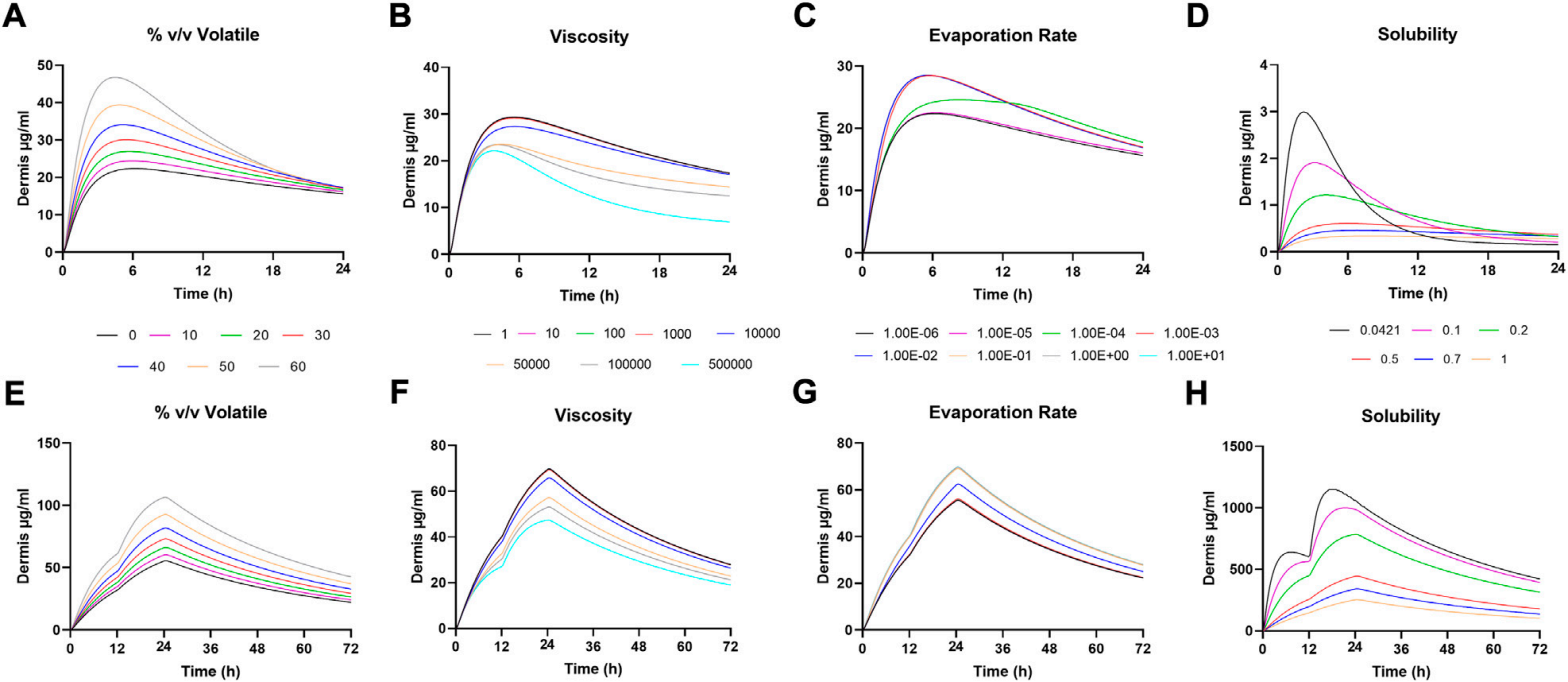
Sensitivity Analysis for **(A-D)** Dermis amounts following IVPT simulation and **(E-H)** Dermis amounts following *in vivo* simulation.

### *In vitro*–*in vivo* extrapolation (IVIVE)

The IVIVE functionality in the Simcyp Simulator takes the optimised IVPT model and restores *in vivo* physiology in the MPML MechDermA model such as dermal blood flow and deeper skin layers, which is then connected with the full PBPK model for simulating *in vivo* ADME. This approach has been described previously (Arora et al., 2022). As there were no data available for DSM intravenous clearance or *in vitro* metabolism, clearance was predicted using pkCSM (Pires et al., 2015). Distribution was predicted using full PBPK method 2 in the Simcyp Simulator (Rodgers and Rowland, 2006).

## Vasoconstriction

To accurately simulate DSM PK *in vivo*, the physiology of the dermis was modified to simulate vasoconstriction. This was achieved by reducing the capillary radius and fraction of capillaries perfused, as described in Table 3. In the depth-resolved dermis model within the MPML MechDermA (Clarke et al., 2018), it is possible to modify capillary radius dynamically in the simulation, and blood flow is automatically recalculated at each time point based on this change. The current simulation assumed capillary radius is reduced two-fold, resulting in an approximately four-fold reduction in the blood flow. In addition, the fraction of perfused capillaries was assumed to be reduced two-fold, resulting in a total blood flow reduction of approximately eight-fold for each individual. The sensitivity of these physiological changes is assessed as follows.

**TABLE 3:** Description of physiological parameters tested in the virtual bioequivalence studies.

Scenario	Description	Comments
Physiology A (default)	Default healthy volunteer with 0.5x capillary radius and fraction perfused, as described earlier	Default assumed physiology; vasoconstriction caused by DSM exposure
Physiology B	Default healthy volunteer without vasoconstriction	To investigate the effect of no simulated vasoconstriction on the VBE assessment
Physiology C	Same as physiology A but with the number of SC layers reduced to 10	To represent the reduced barrier function or skin disease
Physiology D	Same as physiology B but with the number of SC layers reduced to 10	To represent the reduced barrier function or skin disease

### Sensitivity analysis for formulation optimisation

Various formulation-specific parameters were tested for their sensitivity. This included

• API solubility in continuous phase (mg/ml) – range: 0.0421 (DSM water solubility) to 1 mg/ml
• Viscosity (cP) - range: 1 to 500,000 cP
• Maximum amount evaporated (expressed as % v/v volatiles) – range: 0%–60%
• Evaporation rate (ml/h) - range: 1e-6–10 ml/h.

### Virtual bioequivalence assessments

Virtual bioequivalence (VBE) simulations were conducted using the VBE module in the Simcyp Simulator version 21. Non-compartmental analysis and BE assessments were conducted using Phoenix version 8.3 (Certara, NJ).

The formulation parameters listed earlier were also assessed for their sensitivity to cause bio-in-equivalence. VBE simulations were performed by modifying the test formulation parameters within the range to define a safe space (i.e., estimate at which parameter value the formulation becomes bio-in-equivalent). A parallel study design was used due to the lack of data for the inter-occasion variability of skin physiology parameters. This represents a worst-case scenario for the VBE assessment by assuming that the inter-occasion variability is equal to inter-individual variability.

### Power analysis

In order to select a patient number for the safe space analysis, a power analysis was conducted by simulating reference vs. reference (i.e., two identical formulations) for a range of patient numbers. The appropriate number was chosen as the first (to the nearest 10) that shows bioequivalence between two reference products. The limitations of this approach are discussed as follows.

### Dose selection

The patent for DSM spray suggests that 1 spray deposits approximately 2.4 mg of formulation on the skin (Kisak, 2019). This means that the amount applied in the IVPT study is approximately 3.5 sprays per cm^2^ of skin. However, it is unclear if such a high dose is clinically relevant; therefore, the effect of reducing this dose was also tested. The default dose selected for *in vivo* simulations was 1 spray per cm^2^, denoted “dose 3” (see Effect of dose section). The Topicort^®^ Spray label states “apply a thin film twice daily” (Taro, 2021); therefore, two doses were applied, each with a 12-h duration of application to replicate the clinical scenario. The study duration was 72 h to allow the estimation of AUC_inf_.

### Effect of physiology

For each formulation parameter (viscosity, % v/v volatiles, evaporation rate, and solubility), a series of alternative physiological conditions were assessed.

The results of the VBE analyses were used to select upper and lower bound values for each formulation parameter. These were selected as the first value that causes bio-in-equivalence on either side or the maximum/minimum tested value. Both the reference and test patients were assigned the same physiology to assess if recruiting a different patient population for a BE study, for example, with a skin disease, could affect the outcome. The test formulation was then simulated with the upper and lower bounds of formulation parameters for each scenario in Table 3 to assess if physiology interacts with the sensitivity of this parameter and if the outcome of the BE assessment could be affected.

### Effect of dose

The sensitivity of different formulation parameters (viscosity, % v/v volatile, evaporation rate, and solubility) for upper and lower bound values as described earlier was assessed for four different dose amounts, namely, 1) low dose - 1 spray per 10 cm^2^, 2) middle dose - 1 spray per 2 cm^2^, 3) default dose - 1 spray per 1 cm^2^, and 4) IVPT dose - 3.5 sprays per 1 cm^2^_._ Doses 1–3 were chosen to represent a more realistic range for clinical use as compared to the high dose used in the IVPT study.

## Results

### IVPT results

Simulated and observed IVPT results are shown in Figure 1. Amounts in the receptor were well captured other than at the 4-h time point. This appears to be an artefact of the observed data, which was extracted and averaged from several studies, as the 4-h time point does not fit with the surrounding time points on this cumulative mass plot. Local skin amounts were accurately simulated for both the epidermis and dermis.

### Sensitivity analysis

Sensitivity analysis was performed on formulation parameters both *in vitro* and *in vivo*. Figure 2 shows the *in vitro* receptor and *in vivo* plasma, and Figure 3 shows the dermis for both. The solubility of DSM in the formulation showed the highest sensitivity (Figures 2D–H; Figure 3D,H). The lowest sensitivity was for the evaporation rate (Figures 2C,G; Figures 3C,G). The model showed similar sensitivity *in vitro* and *in vivo* for all parameters tested.

## Virtual bioequivalence results

### Power analysis for the virtual bioequivalence study

**FIGURE 4:**
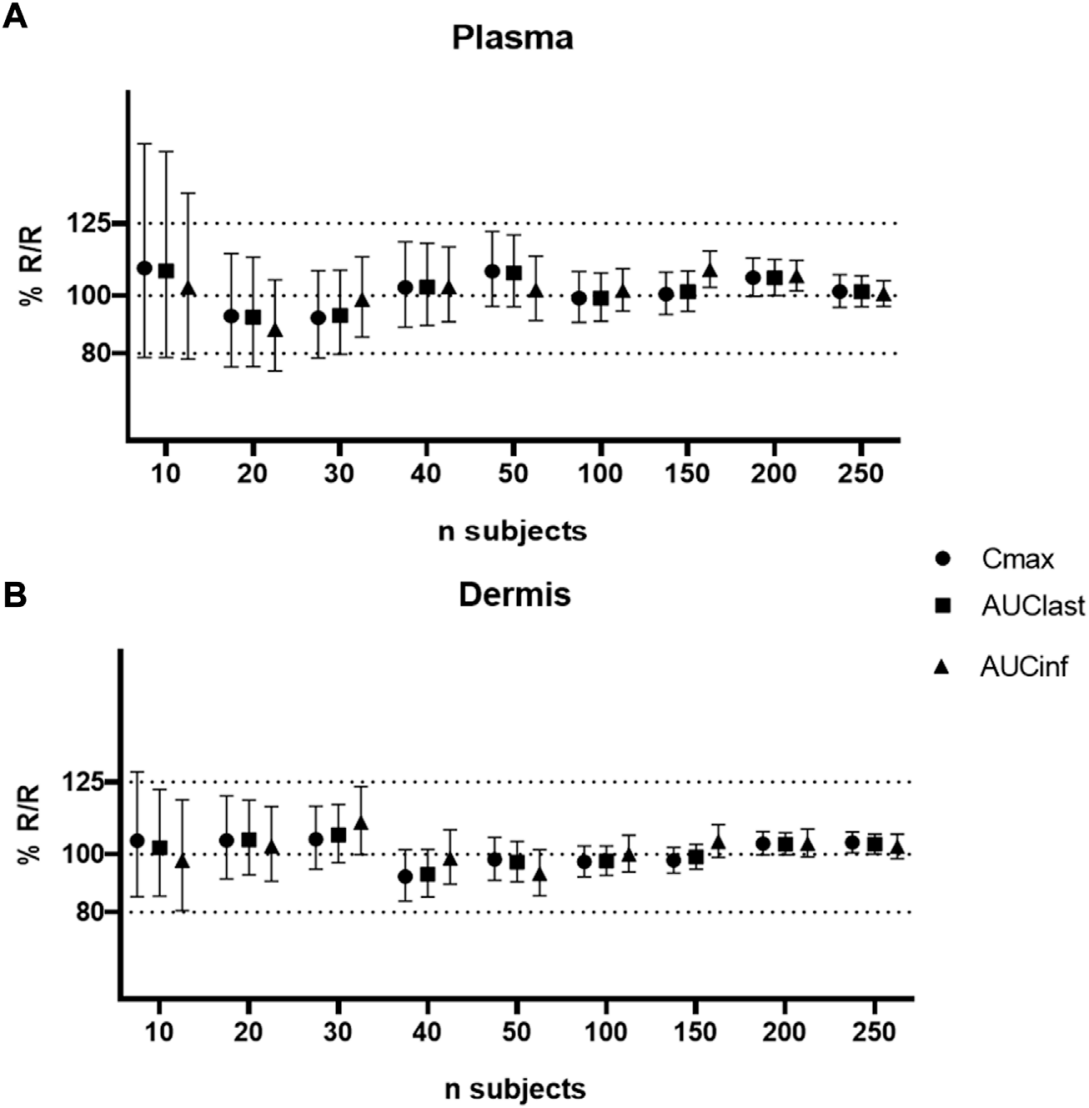
Power analysis for default dosing conditions **(A)** plasma **(B)** dermis.

Study power was assessed for the default dosing conditions of 1 spray per cm^2^ over a 50 cm^2^ application area for a 72-h long study with 12 h as the duration of application for each dose. Figure 4 shows *C*_max_, AUC_last_, and AUC_inf_ ratios for reference vs. reference (% R/R) in plasma and dermis for various patient numbers. Lower patient numbers failed to show BE between the two reference products due to high variability, and the dermal results had narrower confidence intervals than plasma, showing BE with 20 patients per arm. A patient number of 40 was required to show BE in both plasma and dermis, and this was selected as the sample size for further VBE simulations. A total of 40 patients per arm study represent 82, 85, and 90% power in plasma for *C*_max_, AUC_last_, and AUC_inf_, respectively.

### Sensitivity analysis of formulation parameters for the virtual bioequivalence study

A safe space analysis for viscosity, % v/v volatiles, evaporation rate, and solubility in continuous phase was carried out for plasma and dermis concentrations. The effect of modifying these parameters in the test formulation on the relative T/R *C*_max_, AUC_last_, and AUC_inf_ was assessed.

**FIGURE 5:**
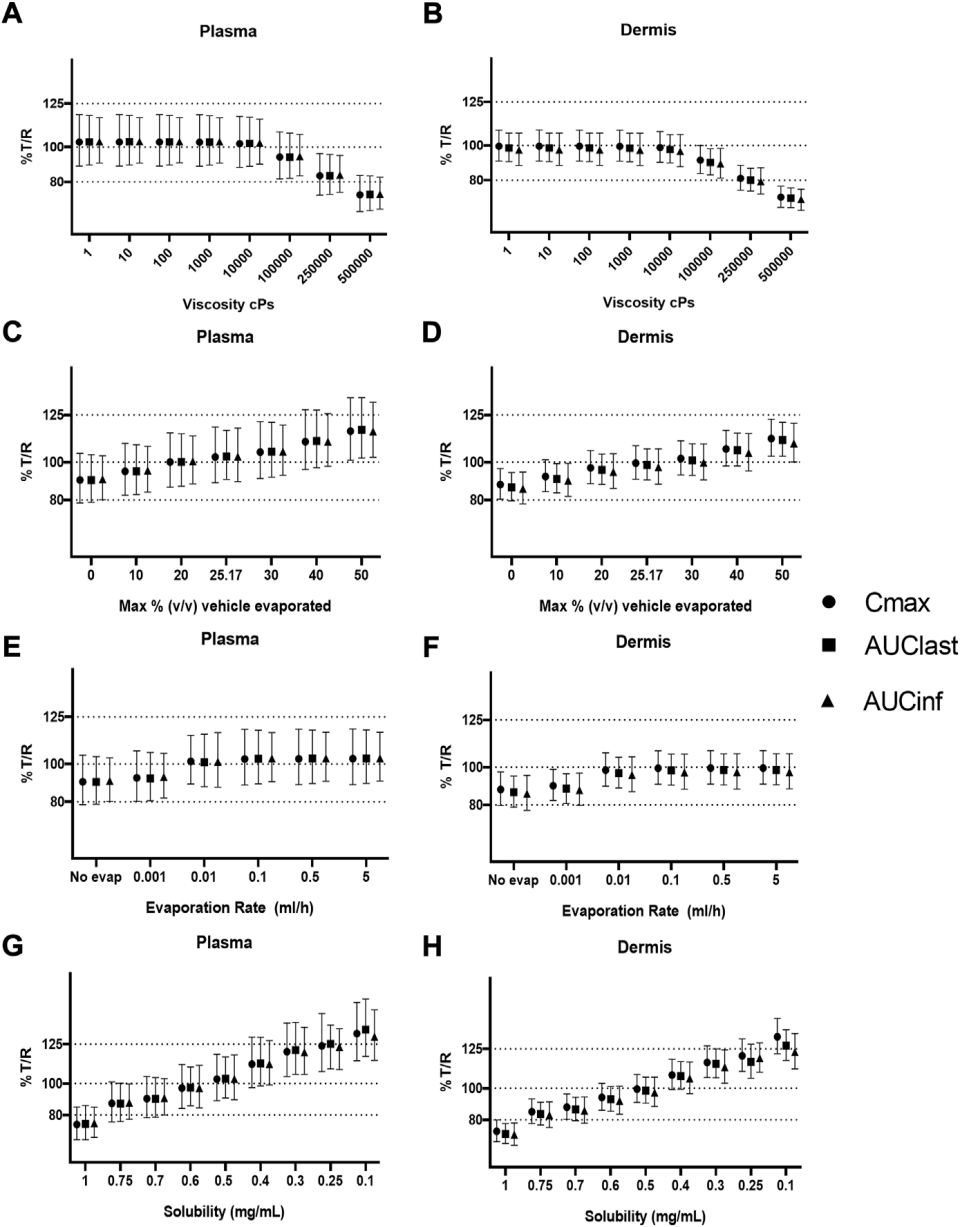
Sensitivity analysis of formulation parameters for virtual bioequivalence; viscosity **(A,B)**, % v/v volatile fraction **(C,D)**, evaporation rate **(E,F)**, and solubility of drug in the continuous phase **(G,H)** for *C*_max_, AUC_last_, and AUC_inf_ in the plasma and dermis.

Figures 5A,B suggest that formulation viscosity has a minimal impact on BE when the test formulation viscosity is varied between 1 and 10,000 cP. The viscosity of 100,000 cP is required before sensitivity is observed, but the *C*_max_ and AUC 90% confidence intervals for the dermis and plasma still fall within the BE criteria. The test formulation fails the BE criteria for both the plasma and dermis when the viscosity is varied and greater than 100,000 cP. Figures 5C,D suggest that the % v/v volatile fraction in the formulation maintains BE in the range of 10 to 30%. However, a value greater than 40% does not meet the BE criteria as the *C*_max_ and AUC 90% confidence intervals for plasma are outside the BE limits (80–125%).

The effect of evaporation rate on BE is depicted in Figures 5E, F. With no evaporation, the test formulation does not meet the BE criteria, whereas a rate between 0.001 and 5 ml/h maintains BE for the reference formulation. Solubility has a significant effect on BE when varied between 0.1 and 1 mg/ml. At lower (0.1–0.4 mg/ml) and higher values (0.7–1 mg/ml) of drug solubility in the continuous phase, the test formulation falls outside of BE limits. The test formulation meets the BE criteria only when the drug solubility in a continuous phase of the test formulation is between approximately 0.4 and 0.7 mg/ml.

### Safe space of formulation parameters in alternate physiology conditions

**FIGURE 6:**
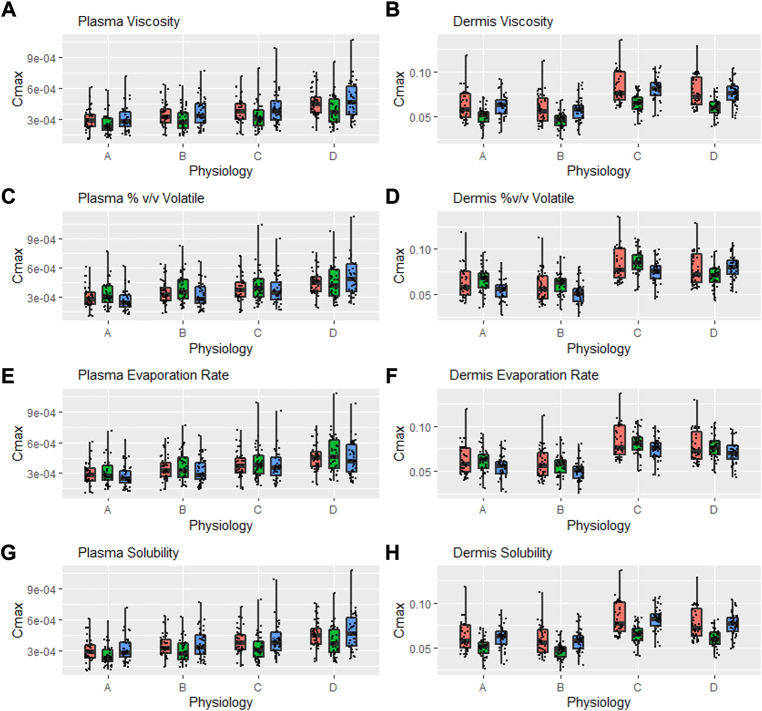
Interaction between formulation parameters and different physiology scenarios for *C*_max_ in plasma and dermis; viscosity **(A,B)**, %(v/v) volatile fraction **(C,D)**, evaporation rate **(E,F)** and solubility of drug in the continuous phase **(G,H)**. Red bars represent RLD parameter value, Green = Upper bound and Blue = Lower bound respectively.

**FIGURE 7:**
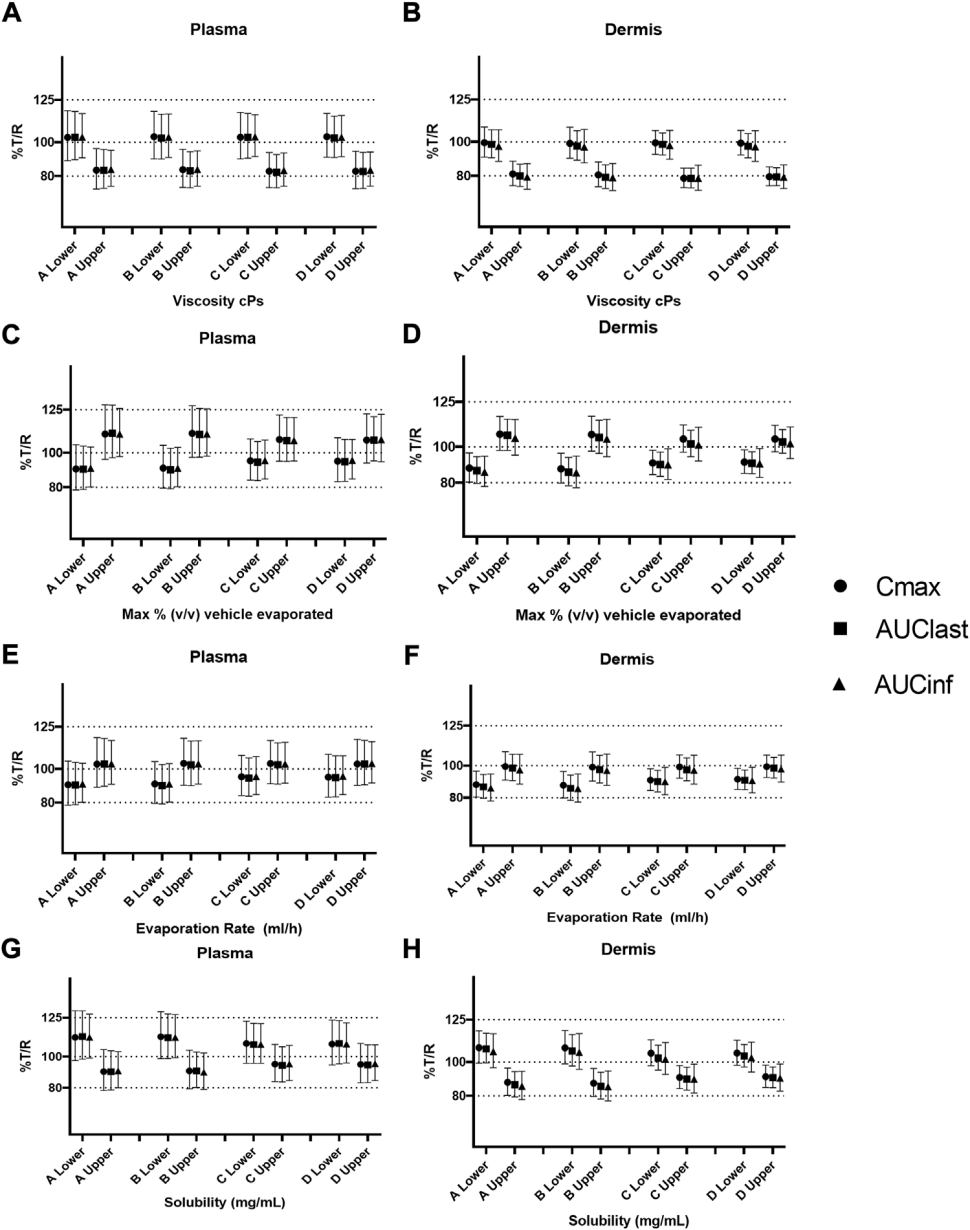
Sensitivity analysis of formulation parameters in alternate physiology conditions; viscosity **(A,B)**, % v/v volatile fraction **(C,D)**, evaporation rate **(E,F)**, and solubility **(G,H)** for physiology scenarios **(A–D)**. The “lower” and “upper” represent the lower and upper bound values for each formulation parameter, respectively.

A further safe space analysis was carried out to assess the effect of different physiology scenarios on formulation parameter sensitivity. A total of four different physiologic conditions were simulated (Table 3), and these have been represented as A, B, C, and D, respectively, in Figures 6 and 7. The results of the VBE analysis in Figure 5 were used to guide the selection of lower and upper bound values for each formulation parameter. The lower and upper bound values selected for viscosity are 1 and 250,000 cP, while for % v/v volatile fraction values they are 0 and 40%, for evaporation rate, they are 0 and 5 ml/h, and for solubility, they are 0.4 and 0.7 mg/ml, respectively The effect of different scenarios on *C*_max_ are shown in Figure 6, and plots for AUC_last_ and AUC_inf_ are shown in the Supplementary Material S1. Physiology scenario B (no vasoconstriction) resulted in a small increase in plasma and dermis *C*_max_; however, it did not have a significant effect on the BE of the tested formulations (Figure 7). Physiology scenarios C and D resulted in a significant increase in *C*_max_ in both plasma and dermis.

The effect of viscosity on *C*_max_ under different physiological scenarios is represented in Figures 6A,B. Figures 7A,B show the effect of different physiology scenarios on the safe space for viscosity. The results show that a change in physiology has no effect on BE limits for both lower and upper bounds of viscosity. At lower values of viscosity (1 cP), *C*_max_ and AUC 90% confidence intervals for plasma and dermis are well within BE limits of 80–125%, while the higher viscosity value (250,000 cP) does not meet BE criteria in all scenarios for both plasma and dermis.

The effect of % v/v volatile fraction on *C*_max_ under different physiological scenarios is represented in Figures 6C,D, while Figures 7C,D show the effect on VBE. At the lower and upper bounds (0% and 40%, respectively), the test formulation does not meet the BE criteria for both physiology A and B in plasma, as *C*_max_ and AUC 90% confidence intervals are outside the BE limits. However, physiology scenarios C and D, which have a reduced number of SC layers, cause formulations at both bounds to move within the BE limits. Similarly, in the dermis, the lower bound does not meet BE criteria for physiology A and B but moves within the criteria when SC barrier function is reduced.

The effect of the evaporation rate on *C*_max_ is represented by Figures 6E,F and Figures 7E,F show the safe space of the evaporation rate with different physiology scenarios. When the barrier function is reduced, the observed effect is similar to that described earlier, and the lower bound evaporation rate (0 ml/h) shows bioequivalence in both the plasma and dermis. The upper bound in this case (5 ml/h) is the same as the reference product and is therefore BE for all physiologies.

The effect of solubility on *C*_max_ is represented by Figures 6G,H. Decreased barrier function causes an increase in *C*_max_ as compared to the default physiology. The effect of tested scenarios on BE is shown in Figures 7G,H. The test formulation falls outside the BE criteria in plasma at the upper bound of solubility (0.7 mg/ml) for physiology scenarios A and B. The lower bound (0.4 mg/ml) falls outside the BE criteria for the plasma and dermis. When tested with physiology scenarios C and D, the model becomes less sensitive to solubility, causing all formulations to become BE.

### Safe space of formulation parameters in different dose amounts

**FIGURE 8:**
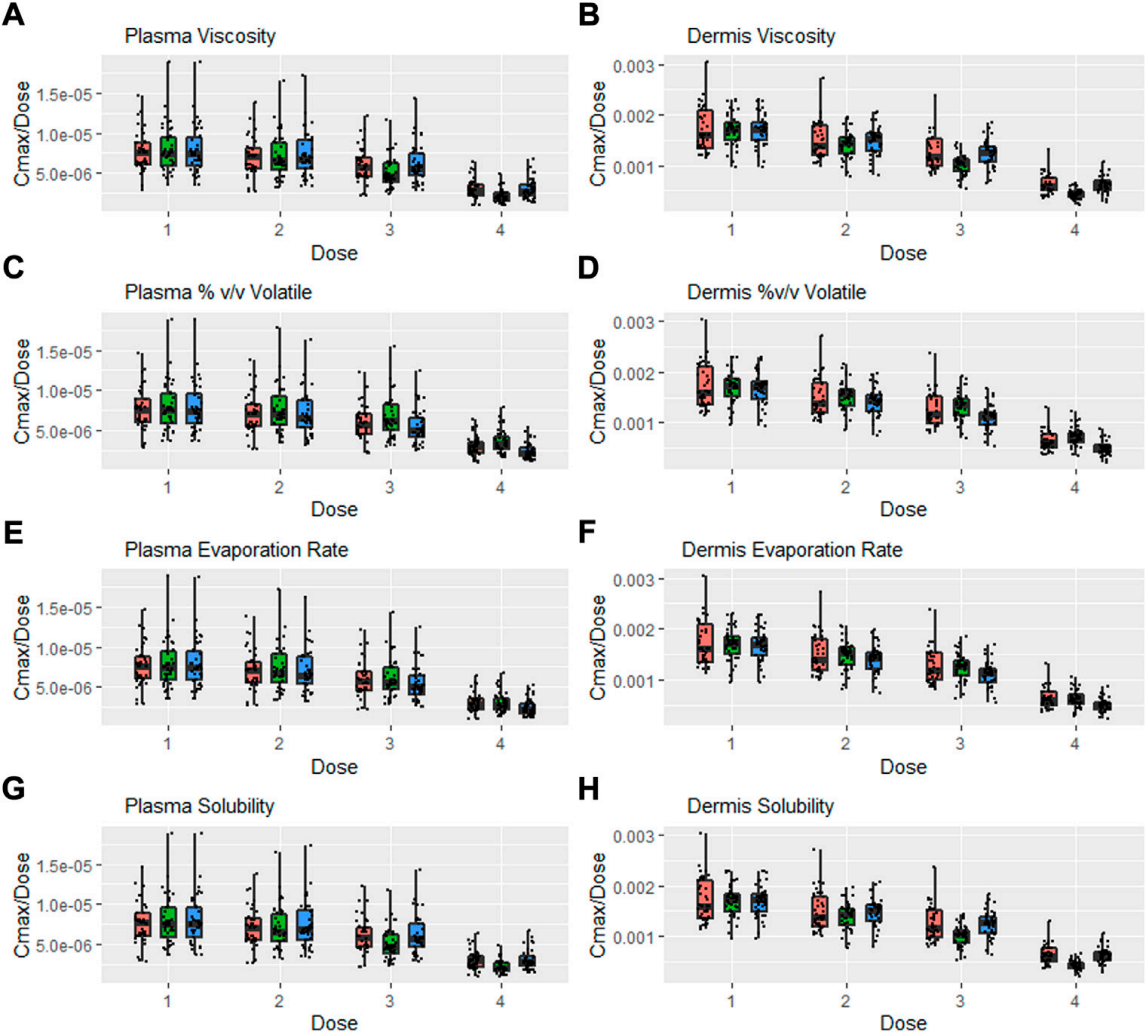
Interaction between formulation parameters and different dosing scenarios, *C*_max_/Dose = *C*_max_/Number of Sprays (i.e., 5, 25, 50, and 3.5 for Dose 1, 2, 3, and 4 respectively); viscosity **(A,B)**, %(v/v) volatile fraction **(C,D)**, evaporation rate **(E,F)** and solubility of drug in the continuous phase **(G,H)**. Red bars represent RLD parameter value, Green = Upper bound and Blue = Lower bound respectively.

A further safe space analysis was carried out to assess the interaction between formulation parameter sensitivity and different dose amounts. Four dose amounts were tested, and Figure 8 represents the *C*_max_ normalised by dose for various formulation parameters after different dose amount applications. Scenario 1 is the lowest dose (one spray per 10 cm^2^), scenario 2 represents one spray per 2 cm^2^, scenario 3 is the default dose (one spray per cm^2^), and scenario 4 is the same dose as the IVPT study, which is approximately 3.5 sprays per cm^2^. Figure 8 shows *C*_max_ normalised with the number of sprays for easier comparison. *C*_max_ was not directly proportional to dose, and this was more evident in the dermis, where the lower dose had a high normalised *C*_max_.

**FIGURE 9:**
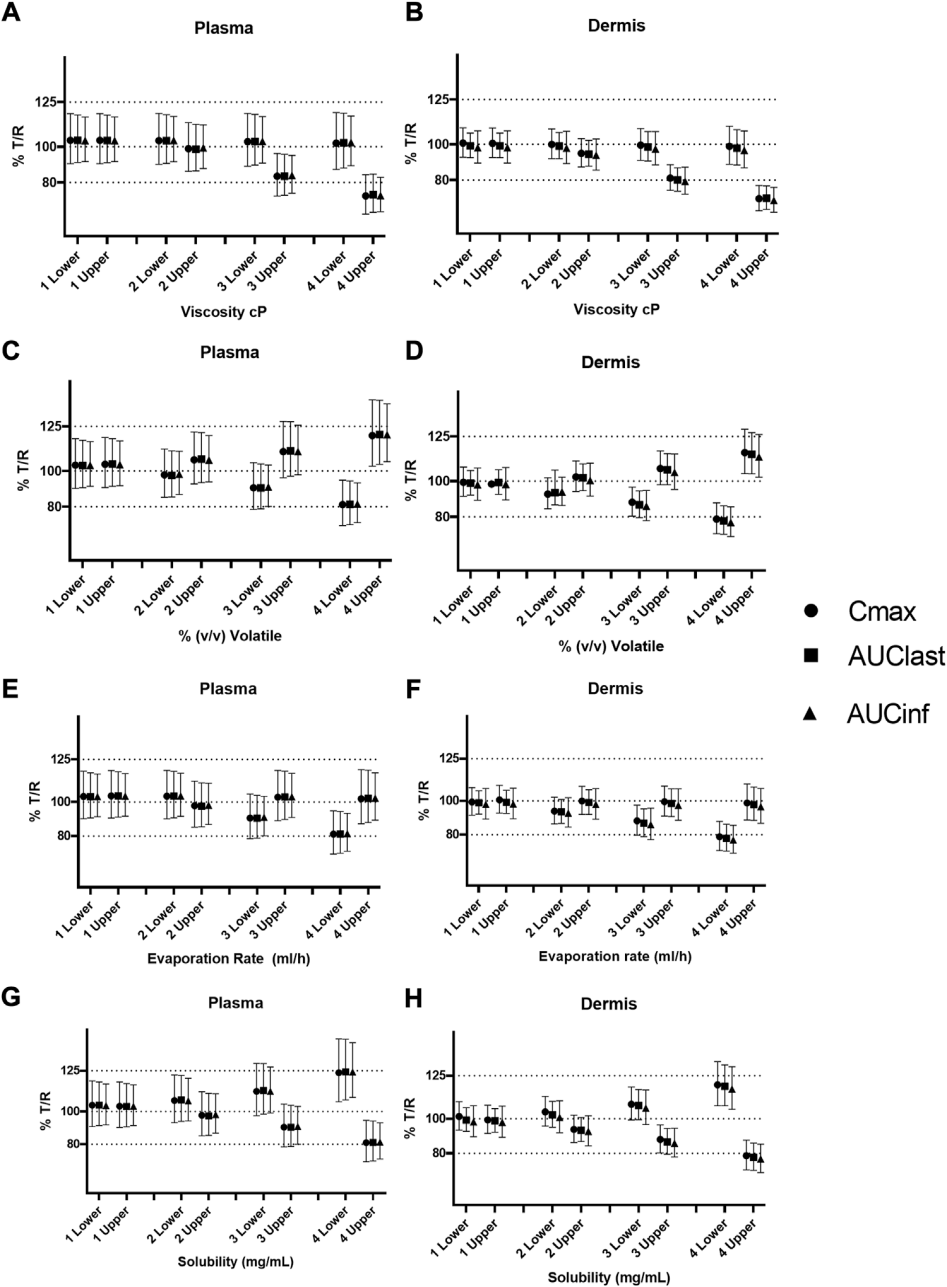
Safe space analysis for formulation parameters at different doses; viscosity **(A,B)**, % v/v volatile fraction **(C,D)**, evaporation rate **(E,F)**, and solubility **(G,H)**. One lower/upper represents the low dose (1 spray per 10 cm^2^); 2 lower/upper represents the middle dose (1spray per 2 cm^2^), and 3 lower/upper represents the default dose (1 spray per 1 cm^2^), respectively, 4 upper/lower represents the dose equivalent to the IVPT study (3.5 sprays per 1 cm^2^).

Sensitivity to all formulation parameters increased with dose. Figure 9 shows the safe space analysis for viscosity (a and b), % v/v volatile (c and d), evaporation rate (e and f), and solubility (g and h) at different dose amounts. The model showed a decreased sensitivity to all formulation parameters with decreasing dose. Upper and lower limits that failed to meet BE criteria for the default dose move within the BE limits for the lower two doses. Similarly, the higher dose from the IVPT study caused some formulations to move further outside the BE criteria. For example, the lower bound for solubility and the upper bound for % v/v volatile, which were BE for the default dose, moved outside the criteria when assessed at the higher dose. The safe space of the % v/v volatile fraction at the different dose amounts is represented by Figures 9C,D. The test formulation for doses 1 and 2 is within the BE limits at both upper and lower values. However, the higher dose causes a divergence of test and reference, causing it to fail at higher doses.

Figures 9E,F represent the safe space for the evaporation rate at the dose amounts. The effect produced is similar to that described earlier, and the lower-bound formulation fails the BE criteria at higher dose amounts.

The effect of solubility is represented in Figures 9G,H. The test formulation fails the BE criteria at the highest dose amount for both upper and lower bounds of solubility.

## Discussion

In the current work, we have developed a PBPK model for Topicort^®^ Spray containing 0.25% DSM. The model was used to investigate the sensitivity and predict a BE safe space for formulation parameters. The only available data for absorption from this formulation were IVPT data from a patent. These data were used to optimise the unknown parameter, the solubility of DSM in the vehicle. Ideally, a second independent dataset would then be used to verify the optimised model, but this was not available for the current formulation. Therefore, caution should be used when drawing specific conclusions about DSM or Topicort^®^ Spray, as the presented models are not sufficiently verified to do so.

The safe space analysis found viscosity to be the least sensitive formulation parameter; a value of greater than 100,000 cP is required to cause bio-in-equivalence. This is because the diffusion of drug within the formulation is not rate limiting at lower viscosities. Considering the viscosity of the reference formulation is estimated to be around 100 cP, the model suggests that viscosity should not be a BE failure point for this formulation. In contrast, solubility of DSM was a highly sensitive parameter, as it has a direct effect on the partitioning behaviour, which is rate limiting for this formulation. The safe space for solubility is very narrow (0.4–0.7 mg/ml), suggesting that this parameter should be tightly controlled to maintain BE. This may be the reason for different absorptions observed in the IVPT results when different oils were substituted for isopropyl myristate (Kisak, 2019).

The % v/v volatile fraction represents the amount of IPA or other volatile solvent in the formulation. The model suggested an increase in the volatile fraction to 40% may cause bio-in-equivalence, but BE was maintained when lowering the volatile fraction, only just failing to meet BE criteria when no evaporation occurs. Similarly, the evaporation rate, which could represent a change in solvent, for example water, which evaporates more slowly, was not sensitive at larger parameter values and just crossed the 80% level when the rate was decreased to such a low level that essentially no evaporation occurred during the study. The simulations with a lower % v/v volatile fraction assumed the volatile fraction was replaced by the tertiary phase constituents, mineral oil, and IPM. The results suggest it may be possible to replace IPM with another volatile solvent with a different evaporation rate and still maintain BE, assuming that this solvent does not cause bio-in-equivalence by significantly affecting another parameter such as solubility.

A limitation of the current approach is that only one formulation parameter was modulated at a time, investigating its safe space while keeping all other conditions the same. In reality, the formulation parameters interact with each other, for example, an increase in viscosity would likely decrease the evaporation rate and a change in the volatile fraction would likely affect solubility. For a formulation with a slower drying rate, it may be necessary to modify solubility over time as the volatile fraction evaporates; however, for the current formulation, evaporation was predicted to proceed rapidly and therefore this was not necessary.

It is possible to assess covariation in the parameters but this would result in too many combinations to plot effectively. A better approach during development may be to test the specific parameters of a series of lead formulations. In some cases, BE is met in the dermis but not in the plasma, and this represents the importance of measuring BE at the site of action rather than using plasma as a surrogate, which may give an inaccurate assessment.

An important factor that is not considered in this example is any direct effect of formulation excipients on the stratum corneum. For example, IPM has been shown to enhance the absorption of some compounds (El Maghraby et al., 2009); therefore, modifying its volume fraction could have effects other than modulation of solubility and evaporation, which would not be captured by the current model. This could be addressed if data for absorption enhancement by different IPM concentrations are available but it is not currently possible to predict this effect *a priori*.

An open question for the bioequivalence of dermatological drug products is whether BE outcomes could be affected by changes in physiology in the patient population, particularly for products that are indicated for a skin disease. The PK of DSM from Topicort^®^ Spray was compared under various physiological scenarios designed to represent a modified barrier function or systemic uptake. The default assumed physiology-simulated vasoconstriction in the dermis caused by the steroid, and this was applied as a static change in the capillary radius and fraction of capillaries perfused. It is possible to use local DSM concentration in the dermis to feedback the extent of vasoconstriction for each individual at any given time point; however, this would require quantification of the pharmacokinetic–pharmacodynamic relationship, which is out of scope for the current work.

Simulations with modified physiology resulted in an increase in *C*_max_ of up to 50% (Figure 6) and an increase in AUC of up to 25% (Supplementary Material S1). This increase was similar between different formulations tested and only resulted in minor changes to the relative BE; however, for formulations that were on the border of the BE acceptance criteria, this resulted in a change of outcome (Figure 7). In this case, a decreased barrier function as represented by the decreased number of SC layers resulted in reduced sensitivity to formulation parameters. Considering the example of solubility in vehicles, which defines the relative affinity between the vehicle and stratum corneum lipids, this parameter is rate limiting at lower values but its sensitivity plateaus at higher values (Figures 3D, H) as the barrier function of the SC is no longer rate limiting at these higher values. By modifying the base permeability of the SC *via* the number of SC layers, a lower value of *K*_sclip:v_ (and therefore a higher value of solubility) is required to reach this plateau. The reason this interaction with physiology is not observed for viscosity is because diffusion within the formulation is independent of any physiology parameter and is still the rate-limiting step at high viscosity values regardless of how fast permeation proceeds in the SC.

The approach of modifying a single physiology parameter here is a simplification, and the number of SC layers and blood flow were chosen as two of the most sensitive physiology parameters in the model that are known to be modified in skin diseases such as psoriasis (Brody, 1963; Nyfors and Rothenborg, 1970; Braverman and Yen, 1977; Alper et al., 2004) and atopic dermatitis (Klemp and Staberg, 1982; White et al., 1987). However, these diseases are also associated with many other physiological changes such as surface pH, corneocyte dimensions, and epidermal thickness (Goldschmidt, 1979; Kashibuchi et al., 2002; Wolberink et al., 2011), which should also be modified in the model to accurately simulate diseased skin. This is an area of ongoing research for the current authors (Polak, 2019), but it is outside the scope of the current document.

The dose used in the IVPT study of ∼10 μL/cm^2^ represents approximately 3.5 sprays of Topicort Spray per cm^2^, which is not realistic for the in-use scenario. Therefore, the effect of different doses on formulation safe space and BE outcomes was assessed. The simulations showed a significant reduction in sensitivity for all formulation parameters with decreasing dose. This is because at lower doses, the formulation is depleted of drug rapidly, meaning only 0.37% of the drug remains in the formulation after 12 h for the lowest dose. Therefore, the effect of the formulation becomes insignificant after a short period of time for the lower dose, as most of the drug has already permeated. In contrast, following the highest dose, 12.3% of DSM remains in the formulation after 12 h.

This study demonstrates a key advantage of incorporating mechanistic models alongside *in vitro* and *in vivo* studies; the ability to investigate alternative dosing scenarios, which in this case were more clinically relevant than the dose used in the IVPT study but would have been challenging or impossible to perform practically. The lower two doses (Scenarios 1 and 2) investigated here represent application rates of ∼1.5 and 0.3 μL/cm^2^, which if applied in an IVPT study would likely result in an incomplete coverage of the skin surface area and would be difficult to quantify; the PBPK model has no such limitations and therefore can be used to complement these studies and add value to the development process.

## Data Availability

The original contributions presented in the study are included in the article/Supplementary Material; further inquiries can be directed to the corresponding author.
