# Supplementary material for: A mechanistic physiologically based model to assess the effect of study design and modified physiology on formulation safe space for virtual bioequivalence of dermatological drug products
Source: Front Pharmacol. 2022 Dec 1;13:1007496. doi: 10.3389/fphar.2022.1007496 (PMC9756572; doi:10.3389/fphar.2022.1007496)
Supplement: Supplementary file 1 [file DataSheet1.docx]

Supplementary Material

## Supplementary Figures


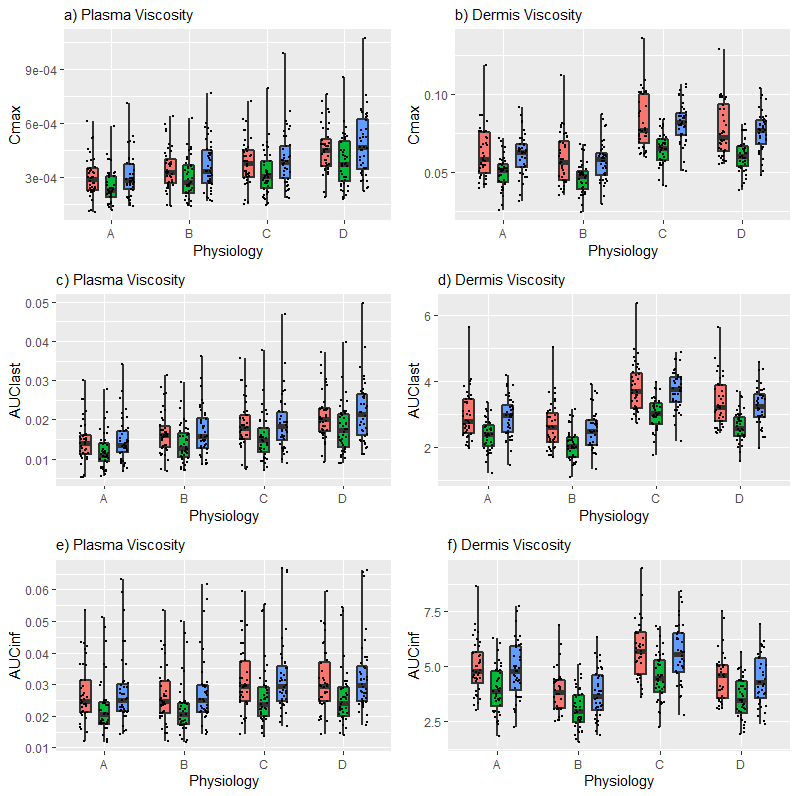


**Supplementary Figure 1.** Cmax, AUClast and AUCinf , for Default (Red), Upper bound (Green) and Lower bound(Blue) for Viscosity in various physiological scenarios.


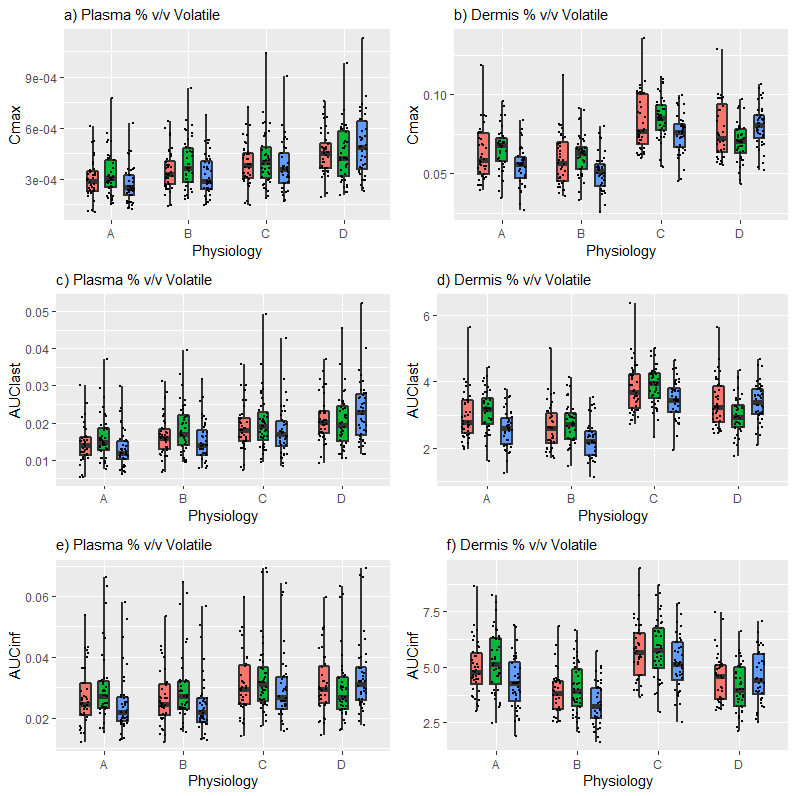


**Supplementary Figure 2.** Cmax, AUClast and AUCinf , for Default (Red), Upper bound (Green) and Lower bound(Blue) for % v/v volatile fraction in various physiological scenarios.


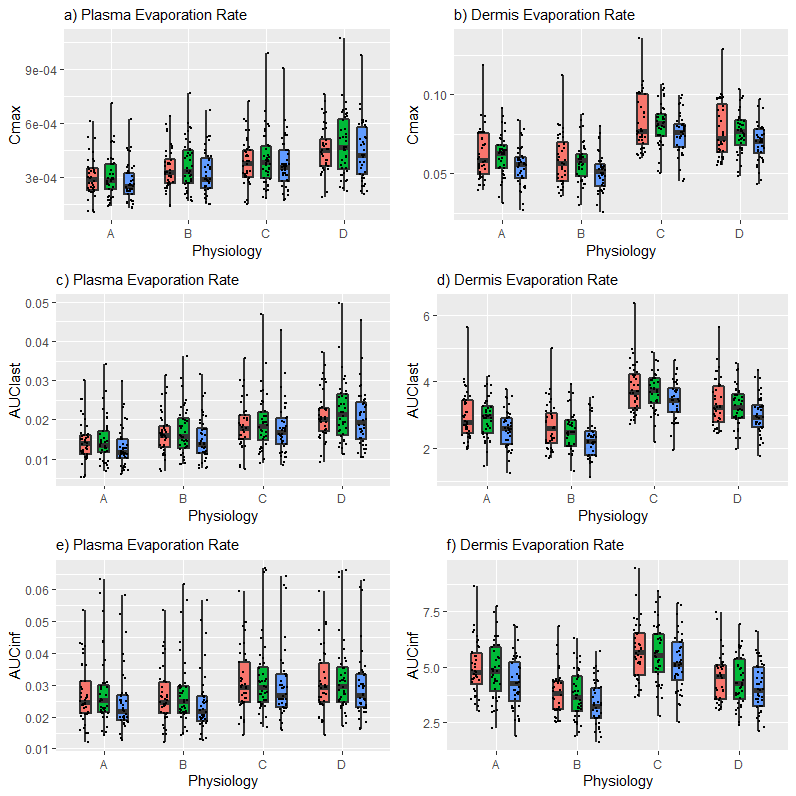


**Supplementary Figure 3.** Cmax, AUClast and AUCinf , for Default (Red), Upper bound (Green) and Lower bound(Blue) for evaporation rate in various physiological scenarios.


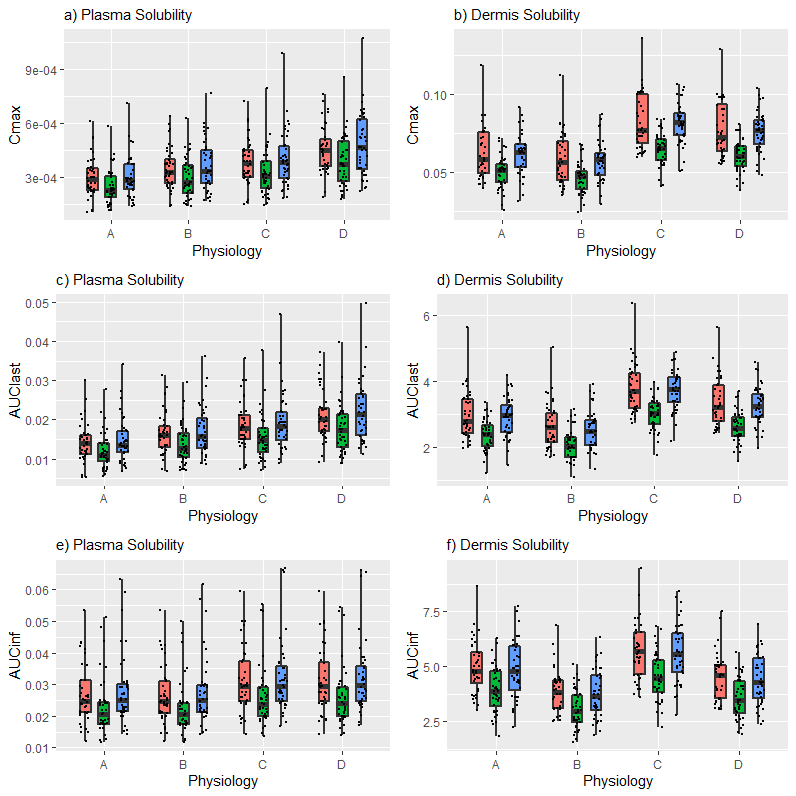


**Supplementary Figure 4.** Cmax, AUClast and AUCinf , for Default (Red), Upper bound (Green) and Lower bound(Blue) for solubility in various physiological scenarios.


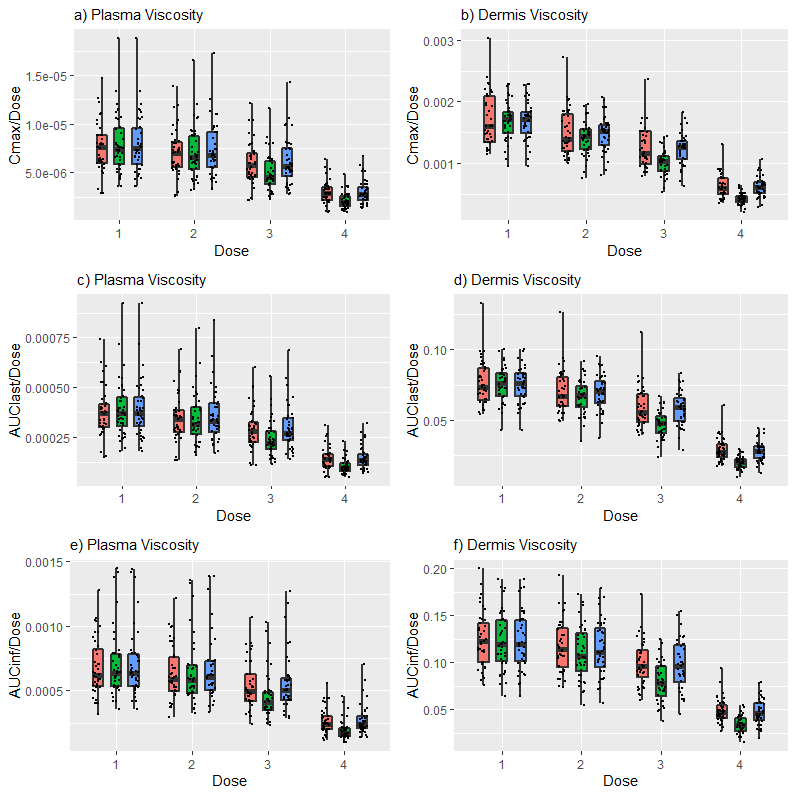


**Supplementary Figure 5.** Cmax/Dose, AUClast/Dose and AUCinf/Dose , for Default (Red), Upper bound (Green) and Lower bound(Blue) for Viscosity following difference dose scenarios


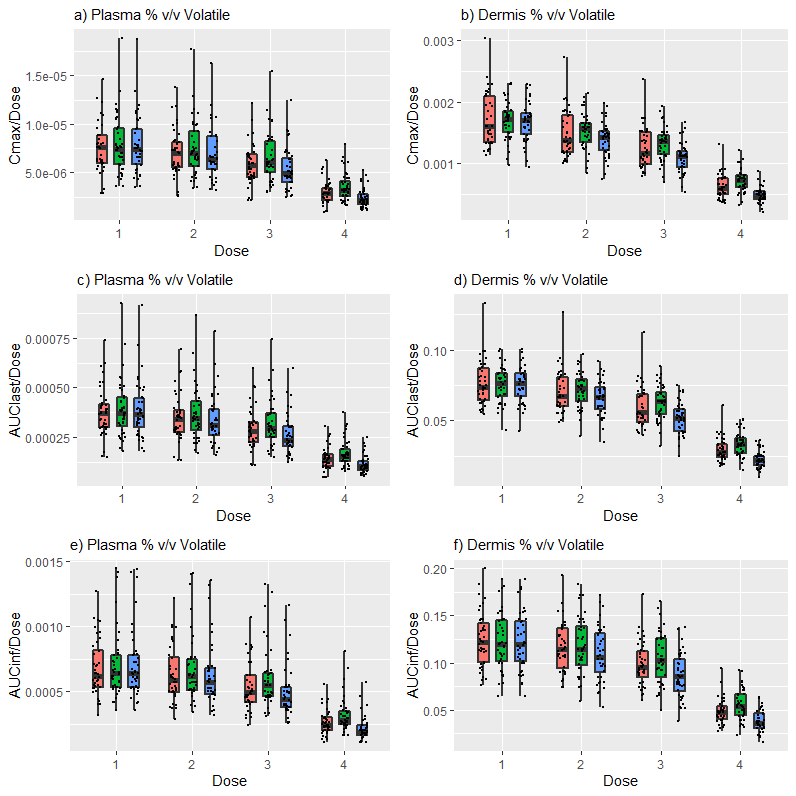


**Supplementary Figure 5.** Cmax/Dose, AUClast/Dose and AUCinf/Dose , for Default (Red), Upper bound (Green) and Lower bound(Blue) for %v/v volatile fraction following difference dose scenarios


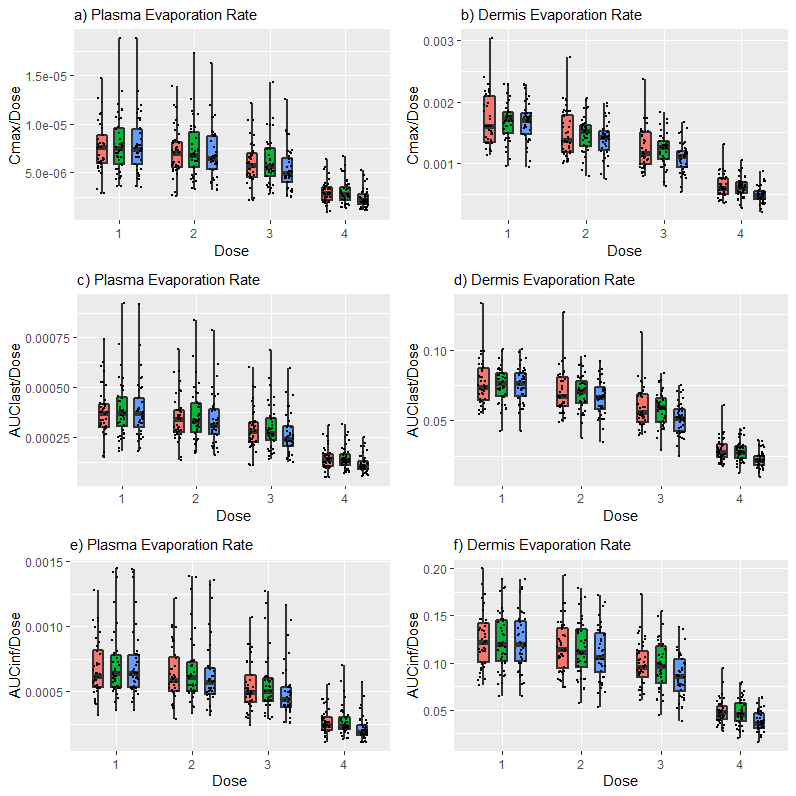


**Supplementary Figure 5.** Cmax/Dose, AUClast/Dose and AUCinf/Dose , for Default (Red), Upper bound (Green) and Lower bound(Blue) for evaporation rate following difference dose scenarios


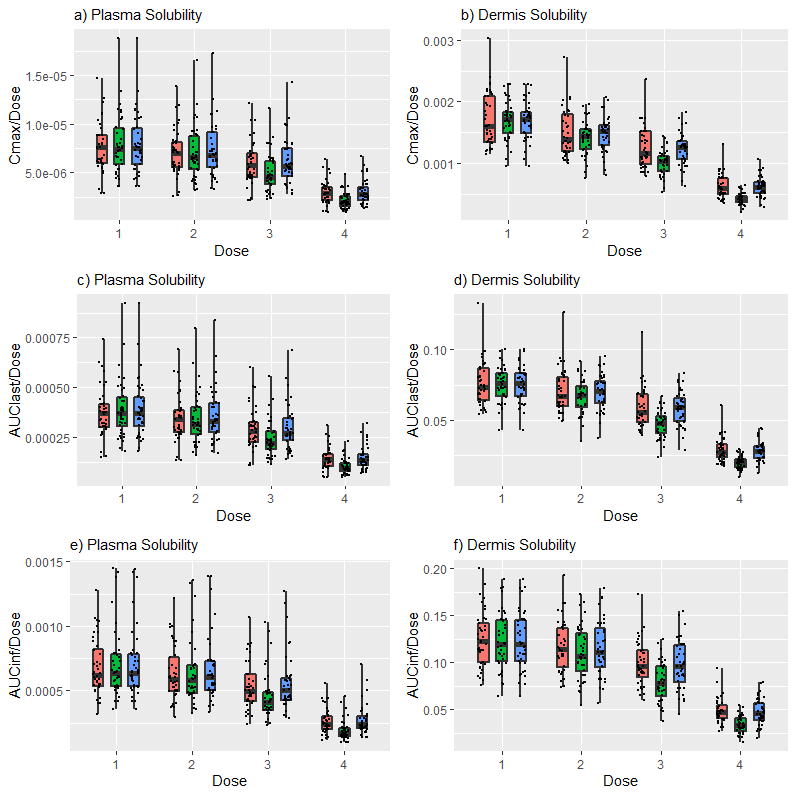


**Supplementary Figure 5.** Cmax/Dose, AUClast/Dose and AUCinf/Dose , for Default (Red), Upper bound (Green) and Lower bound(Blue) for solubility following difference dose scenarios
